# Retrospective Case-Control Study on the Effect of In-Person Physical Therapy With Remote Therapeutic Monitoring on Functional Outcomes and Plan of Care Adherence Amongst Individuals With Musculoskeletal Conditions

**DOI:** 10.1016/j.arrct.2025.100466

**Published:** 2025-05-24

**Authors:** Timothy Marshall, Andrew Goldman, Robert Lyles, M. Jake Grundstein, Negar Ahmadian, Thomas A. Koc, Marc Gruner

**Affiliations:** a Ivy Rehab Network, White Plains, NY; b Limber Health, Bethesda, MD; c Kean University, Union, NJ

**Keywords:** Musculoskeletal diseases, Rehabilitation, Technology, Therapeutics

## Abstract

**Objective:**

To evaluate the efficacy of in-person physical therapy (PT) coupled with remote therapeutic monitoring (RTM) compared to in-person PT only on patient outcomes and care delivery.

**Design:**

A case-control study

**Setting:**

95 private practice physical therapy clinics. RTM is delivered at home via a mobile application.

**Participants:**

Inclusion criteria included: (1) Adults ≥18 years of age, (2) musculoskeletal diagnosis, (3) clinician-prescribed PT, (4) at least 2 outcome measures. Patients who met the inclusion criteria were enrolled in RTM. A control group was generated using 3:1 matching based on: age, sex, case type, and intake patient-reported outcome score. Three hundred and six cases for the in-person PT + RTM group (N = 306) and 918 (N = 918) controls were identified.

**Interventions:**

RTM Patients were enrolled in a home exercise program administered through a mobile application, with digital exercise therapy videos and care navigation support. Both RTM and control patients were enrolled in in-person PT.

**Main Outcome Measures:**

Achieving the discharge functional status score as measured by the binary yes/no Functional Status Benchmark.

**Results:**

A significantly greater proportion of PT + RTM patients achieved the Functional Statue Benchmark (72%) compared to the control group (63%, *P*=.004). A statistically greater proportion of PT + RTM patients attended more than 2 visits per week (36%) compared to the control group (24%, *P*<.001). When controlling all variables, RTM participation was a significant predictor of achieving the discharge functional status score as measured by the binary yes/no Functional Status Benchmark (adjusted odds ratio, 1.53; 95% confidence interval, 1.04-2.22).

**Conclusions:**

The inclusion of RTM with in-person PT facilitated better patient engagement and patient-reported outcomes compared to in-person PT only.

Estimates suggest that musculoskeletal (MSK) conditions, which can be defined as conditions and/or injuries that affect, muscles, joints, and bones that cause pain and loss of function, impacting quality of life, burden more than half of Americans over the age of 18, a number that is expected to increase with the aging American population.^1^^,^^2^ The effect of MSK conditions is significant; aside from the detriment to patients’ quality of life and functionality, they also cost the United States (US) health care system an estimated $420 billion annually. This expense outweighs that of any other chronic condition, leading researchers to examine the most clinically effective and cost-efficient interventions for those with MSK conditions.^3^ Research suggests that physical therapy (PT) and home exercise programs are not only effective interventions for reducing MSK-related functional deficits and pain but also are relatively low cost when compared to traditional approaches to care. Furthermore, it has been shown that PT for the treatment and management of MSK-related conditions may also support a significant reduction in downstream health utilization and spending.^4^^,^^5^ One study confirmed that early and adherent to PT can reduce the need for subsequent injections, medications, imaging, and surgeries by as much as 60%, contributing to significant cost savings if the entire affected population is considered.^6^

Unfortunately, patients may encounter barriers to remaining adherent to PT. Kessels^7^ showed as much as 80% of information told to patients in the clinic is immediately forgotten, including key plan of care considerations, and nuances specific to the recommended treatment plan. Additionally, up to 70% of patients are noncompliant with their prescribed home exercise program, further limiting the potential effect and effectiveness of PT, given that around 80% of health outcomes are determined by out-of-clinic factors.^8^^,^^9^ In recent years, digital technologies have surfaced as a potential solution to the barriers surrounding Physical Therapy access and patient adherence and engagement. The COVID-19 pandemic has expedited the use of these remote health platforms, including Digital Exercise Therapy Applications (DETAs).^10^ Early studies have shown improvements in clinical outcomes such as pain, function, disability, and mental health for patients using digital rehabilitation programs, including DETAs.^11^^,^^12^ Areias et al^13^ found not only a significant reduction in pain, but a superior improvement in function for patients who participated in a digital care program as compared to nonparticipants, suggesting the potential of these solutions to increase compliance with home exercise programs. Although these results are promising, digital-only care pathways present clear disadvantages. The lack of physical contact within the model may negatively affect the evaluation and treatment of patients, limiting opportunities to adequately exclude red flags, perform special tests to diagnose the cause of movement impairments and limitations, collect objective data, and implement manual treatment strategies.^14^

Remote therapeutic monitoring (RTM) has emerged as a supplement to the traditional PT care model, and an alternative to strict in-clinic or digital-only pathways. This hybrid model of care augments in-clinic rehabilitation with the use of a remote platform that can administer guided exercise videos, collect patient-reported outcomes, and provide virtual support. Coupled with the emergence of Current Procedural Terminology Codes for RTM in January of 2022, RTM has opened the door for providers to begin tracking patient adherence, engagement, and progress with digital home exercise programs.^15^ Emerging data from qualified digital MSK health applications can help determine whether the inclusion of RTM with traditional PT care plans can improve care, and to what extent.

The purpose of this study is to evaluate the efficacy of in-person PT coupled with RTM (PT+RTM) compared to traditional in-person PT (PT only) on patient outcomes and care delivery.

## Methods

This is a retrospective case-control study of electronic medical record data approved by the Kean University Institutional Review Board (IRB 23-011102). Informed consent was waived because of the study design. All data were deidentified in compliance with the US Health Insurance Portability and Accountability Act.

### Participants and data collection

A large national PT group in the US with clinics across 15+ states partnered with an RTM vendor that provided digital exercise therapy via an application in July 2022 to offer RTM to patients with MSK diagnoses. RTM was offered to patients who sought treatment within the national PT groups, 95 clinics in the New Jersey area, and over 300 physical therapists in total. Patients were eligible for RTM based on case type and insurance, identified in the electronic medical record system. To enroll a patient in RTM, the treating physical therapist educated the patient on the use of the DETAs (software as a medical device), assisted with the application setup, and developed a comprehensive home exercise program for the patient to access through the application.

The DETAs were developed with input from physiatrists and physical therapists to provide personalized, evidence-based home exercise programs as well as education and self-management strategies. The DETA offers detailed instructions for each exercise, educational materials, session reminders, and gamification techniques to enhance engagement.^12^ Once the patient was introduced to the application, they were encouraged to track their home exercise program via the DETA to provide useful data to the therapist between in-clinic visits, improve therapy adherence and engagement, and inform the plan of care. For this study, adherence is defined as the percentage of scheduled in-person physical therapy appointments that were kept visits (arrival rate) and engagement is the number of times a patient keeps an appointment per week. Care Navigators managed through the RTM vendor, were available to the patients involved throughout their care plan to address questions regarding their home exercise program, provide adherence and engagement encouragement, review data, and offer technological support specific to the DETA. Furthermore, the care navigation staff frequently updated the treatment of physical therapists on their respective patient’s progress between in-clinic visits. All involved care navigation staff members were licensed PT assistants trained in motivational interviewing and coaching.

After patients attended their first visit and initiated care, information from the encounter was documented within the electronic medical record.

### Measures

Patient-reported outcomes were collected using the Patient Inquiry software developed by Focus on Therapeutic Outcomes (FOTO), a Net Health Company. FOTO captures a patient’s perceived level of function and ability to participate in activities at work, around the home, and in recreation. These dimensions of function and participation are consistent with the World Health Organization’s International Classification of Functioning, Disability, and Health. Scores are reported on a linear metric from 0 to 100, with higher scores indicating better function.^16^ FOTO provides an initial score that represents the patient’s baseline function as well as a discharge functional status (FS) score, a risk-adjusted score that the patient is projected to achieve by the end of therapy.^17^

To be eligible for analysis, patients must have been ≥18 years of age and received care for a MSK-related issue, with an episode of care discharged between February 27, 2022 and February 27, 2023. The lower age limits were selected because of the protected status of those patient populations. Patients were only included in the analysis if they were administered at least 2 outcomes: 1 outcome during the initial evaluation and 1 outcome status at any time afterward. Any patients containing missing data for any variable of interest were deleted from the analysis. All patients who met the inclusion criteria were matched to the National Physical Therapy group’s outcome data using the patient’s medical record number and case code.

The following data were extracted from the files of eligible patients: age (y), sex, insurance type (commercial, federal, or “other” to include self-pay, receiving workers’ compensation, automobile insurance), case type, initial FOTO score, total number of visits, duration of care (measured in d), average number of visits per week, billable units per visit, arrival rate (calculated as a percentage of scheduled appointments attended by the patient), key provider visit rate (calculated as a percentage of visits in which the patient’s primary therapist administered their care), worst pain at intake (on a numeric rating scale of 0-10), complexity (calculated by the number of International Classification of Diseases-10 codes in the patient’s chart), RTM participation, and discharge reason. Adherence was measured by the percentage of scheduled visits that were kept visits (arrival rate), whereas engagement was measured as the frequency of kept visits per week. The main outcome, Functional Status Benchmark Achieved, was a binary yes/no variable that indicates if the patient’s latest functional score matched or exceeded the discharge functional status score.

### Case-control selection

Cases were identified as patients who met the inclusion criteria and enrolled in RTM. Patient case-level data for closed/discharged episodes between February 27, 2022 and February 27, 2023 were extracted from the National Physical Therapy provider’s data repository. The case-level data had been previously curated into a ‘case-level’ table that included the data points necessary for this study. The data were exported as a csv.file. Using the patient’s unique number in Ivy’s case data as an identifier, we then inner joined to a data extract provided by the RTM vendor in to isolate patients who had received d RTM. Patients that matched to the RTM Vendors data were included in the study in the In-Person + RTM group. Patients who completed < 3 RTM sessions were excluded from the In-Person + RTM group.

**Fig 1:**
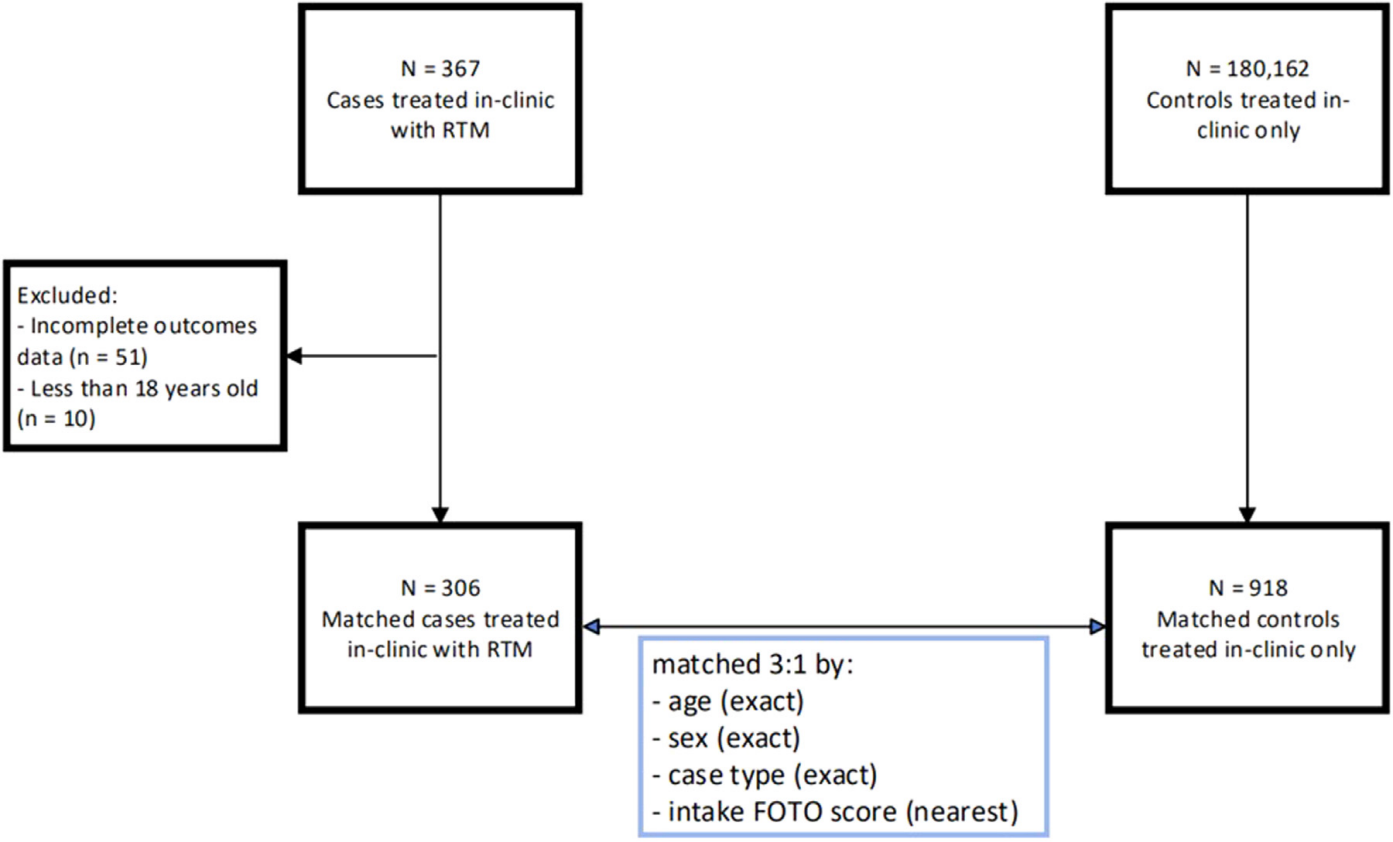
Flow diagram for case-control selection and matching process.

A control group was generated using 3:1 matching based on: (1) age (exact), (2) sex (exact), (3) case type (exact), and (4) intake FOTO score (nearest). For intake/initial FOTO score, controls were matched using one-to-one nearest neighbor propensity scores estimated through logistic regression.^18^^,^^19^ Discharge reason was not a category for matching because (1) there are no standardized SOPs/criteria/definitions for the categories; and (2) the authors hoped to analyze any potential effect of RTM on self-discharge rate. Matched subclasses were checked to ensure that patients with multiple episodes of care were not self-matched and that no patient served as both a control and a case. After matching, all standardized mean differences were below 0.7, and 306 cases and 918 controls were identified for a total N = 1244 (fig 1).

### Statistical analyses

Analyses were conducted using R 4.2.2.^20^^, a^ Univariate and bivariate tables were generated using the gtsummary^21^ and huxtable^22^ packages. Baseline differences between FS benchmark groups were tested for significance at the 0.05 level using Pearson’s chi-square or Wilcoxon rank sum test, as appropriate. The survival package^23^ was used to create a conditional logistic regression model, calculating adjusted odds ratios (aORs) to investigate how each independent variable affected the likelihood of achieving the discharge functional status score as measured by the binary yes/no Functional Status Benchmark.^24^^,^^25^

## Results

### Participants

The demographic characteristics between PT + RTM patients (n=306) and controls who received PT only (n=918) were matched, thus no significant differences were observed between the characteristics (table 1). Significant differences were observed in the insurance type category, as a greater proportion of patients in the control group were classified as “Other” (9.6%), compared to patients in the PT + RTM group (0.3%, *P*<.001). The patients in the PT + RTM group averaged 88.15 minutes (SD, 59.87) across their episodes of care.

**Table 1:** Patient characteristics.

	Overall			
Characteristic	N = 1224	Control (PT Only), N = 918	PT + RTM, N = 306	*P* Value
Age, mean (SD)	61.93 (14.30)	61.93 (14.31)	61.92 (14.30)	.970⁎
Age (recoded), n (%)				.991†
≤18 y	0 (0%)	0 (0%)	0 (0%)	
19-28 y	45 (3.7%)	34 (3.7%)	11 (3.6%)	
29-45 y	136 (11%)	101 (11%)	35 (11%)	
46-64 y	364 (30%)	275 (30%)	89 (29%)	
65+ y	679 (55%)	508 (55%)	171 (56%)	
Sex, n (%)				>.999‡
Male	468 (38%)	351 (38%)	117 (38%)	
Female	756 (62%)	567 (62%)	189 (62%)	
Insurance type, n (%)				<.001⁎
Commercial	501 (41%)	365 (40%)	136 (44%)	
Federal	634 (52%)	465 (51%)	169 (55%)	
Other	89 (7.3%)	88 (9.6%)	1 (0.3%)	
Case type, n (%)				>.999†
Ankle	68 (5.6%)	51 (5.6%)	17 (5.6%)	
Back	560 (46%)	420 (46%)	140 (46%)	
Elbow	12 (1.0%)	9 (1.0%)	3 (1.0%)	
Foot	32 (2.6%)	24 (2.6%)	8 (2.6%)	
General	4 (0.3%)	3 (0.3%)	1 (0.3%)	
Hip	112 (9.2%)	84 (9.2%)	28 (9.2%)	
Knee	164 (13%)	123 (13%)	41 (13%)	
Lower extremity	28 (2.3%)	21 (2.3%)	7 (2.3%)	
Neck	80 (6.5%)	60 (6.5%)	20 (6.5%)	
Shoulder	152 (12%)	114 (12%)	38 (12%)	
Upper extremity	8 (0.7%)	6 (0.7%)	2 (0.7%)	
Wrist	4 (0.3%)	3 (0.3%)	1 (0.3%)	
Initial FOTO score, mean (SD)	48.46 (14.25)	48.45 (14.02)	48.50 (14.96)	.922⁎

⁎ Wilcoxon rank sum test.

† Fisher exact test.

‡ Pearson’s chi-square test.

### Outcomes

A significantly greater proportion of PT + RTM patients achieved the discharge functional status score as measured by the binary yes/no Functional Status Benchmark (72%) compared to controls who received PT only (63%, *P*=.004) (table 2). Patients in the PT + RTM also demonstrated significantly greater engagement as noted by a greater average number of visits per week category, as a greater proportion of PT + RTM patients had >2 visits per week (36%) compared to controls (24%, *P*<.001). No significant differences were observed in the number of total visits (*P*=.672), arrival rate (*P*=.214), key provider visit rate (*P*=.078), complexity (*P*=.795), worst pain at intake (*P*>.999), and discharge reason (*P*=.507) categories.

**Table 2:** Patient outcomes.

	Overall			
Characteristic	N = 1224	Control (PT Only), N = 918	Intervention PT + RTM, N = 306	*P* Value
**Functional status benchmark, n (%)**				.004
Functional status benchmark missed	424 (35%)	339 (37%)	85 (28%)	
Functional status benchmark achieved	800 (65%)	579 (63%)	221 (72%)	
**Total visits (recoded), n (%)**				.672
≤5	79 (6.5%)	63 (6.9%)	16 (5.2%)	
6-9	189 (15%)	145 (16%)	44 (14%)	
10-14	299 (24%)	223 (24%)	76 (25%)	
15+	657 (54%)	487 (53%)	170 (56%)	
**Duration of care (d), mean (SD)**	70.19 (47.84)	71.65 (50.57)	65.80 (38.24)	.681
**Visits per wk (recoded), n (%)**				<.001
≤2	891 (73%)	694 (76%)	197 (64%)	
>2	333 (27%)	224 (24%)	109 (36%)	
**Arrival rate (recoded), n (%)**				.214
<80%	373 (30%)	282 (31%)	91 (30%)	
80-89%	330 (27%)	236 (26%)	94 (31%)	
≥90%	521 (43%)	400 (44%)	121 (40%)	
**Key provider visit rate (recoded), n (%)**				.078
0-49%	147 (12%)	117 (13%)	30 (9.8%)	
50-69%	310 (25%)	219 (24%)	91 (30%)	
≥70%	767 (63%)	582 (63%)	185 (60%)	
**Complexity (# of ICD10 codes, recoded), n (%)**				.795
≤2	891 (73%)	670 (73%)	221 (72%)	
>2	333 (27%)	248 (27%)	85 (28%)	
**Worst pain at intake, n (%)**				.996
≤6	358 (38%)	264 (38%)	94 (38%)	
>6	590 (62%)	435 (62%)	155 (62%)	
Unknown	276	219	57	
**Discharge reason, n (%)**				.507
DC by provider	689 (56%)	508 (55%)	181 (59%)	
Self-DC	239 (20%)	183 (20%)	56 (18%)	
Other DC reason	296 (24%)	227 (25%)	69 (23%)	

Pearson’s chi-square test.

A conditional logistic regression model was created to assess the main effect of RTM on the main outcome (Functional Status Benchmark) while controlling for the other variables included in the model. When controlling all variables, which included insurance type, units per visit, total visits, visits per week, arrival rate, and worst pain at intake, only RTM participation was a significant predictor of achieving the discharge functional status score as measured by the binary yes/no Functional Status Benchmark outcome (aOR, 1.53; 95% CI, 1.04-2.22) (table 3). Compared to patients who attended 80%-89% of their scheduled visits, patients who attended ≥90% of their scheduled visits had greater odds of achieving the discharge functional status score as measured by the binary yes/no Functional Status Benchmark (aOR, 2.29; 95% CI, 1.47-3.58). Insurance type, total visits, visits per week, key provider visit rate, and worst pain at intake were not significant predictors of achieving the discharge functional status score as measured by the binary yes/no Functional Status Benchmark (table 3).

**Table 3:** Matched data: adjusted model predicting functional status benchmark achievement.

Characteristic	OR (95% CI)	*P* Value
**RTM participation**		
Control (PT only)	—	
PT + RTM	1.52 (1.04-2.22)	.032
**Insurance type**		
Commercial	—	
Federal	1.45 (0.86-2.47)	.166
Other	0.40 (0.14-1.13)	.085
**Units per visit (recoded)**		
≤4	—	
>4	1.25 (0.86-1.84)	.245
**Total visits (recoded)**		
10-14	—	
≤5	0.93 (0.45-1.94)	.852
6-9	1.34 (0.76-2.35)	.308
15+	1.48 (0.96-2.28)	.077
**Visits per week (recoded)**		
≤2	—	
>2	0.94 (0.60-1.47)	.784
**Arrival rate (recoded)**		
80-89%	—	
<80%	0.85 (0.52-1.39)	.520
≥90%	2.29 (1.47-3.58)	<.001
**Key provider visit rate (recoded)**		
50-69%	—	
0-49%	0.53 (0.28-1.02)	.056
≥70%	1.11 (0.73-1.69)	.635
**Worst pain at intake**		
≤6	—	
>6	0.89 (0.61-1.29)	.526

Abbreviations: OR, odds ratio.

## Discussion

MSK conditions are debilitating and continue to be a significant driver of US health care spending. The effectiveness of PT interventions for MSK conditions can be enhanced by maximizing adherence and engagement to treatment. Interventions such as RTM have the potential to overcome barriers to care and improve adherence and engagement, thereby optimizing outcomes.^26^, ^27^ This retrospective study aimed to assess the effect of a DETA inclusive of RTM services combined with in-person PT on the engagement and clinical outcomes of patients with MSK diagnoses, compared to conventional in-person-only PT. Functional status and adherence were both higher among the group who received the combined PT RTM approach compared to the control group after the intervention.

The results of the study suggest that patients are more likely to achieve their projected discharge functional status score as measured by the binary yes/no Functional Status Benchmark when in-person care is paired with RTM. Additionally, patients whose plan of care included RTM were more likely to attend 2 in-person visits per week, compared to those patients enrolled in the in-person-only PT group. This is particularly noteworthy because insurance type, total visits, visits per week, key provider visit rate, and worst pain at intake were not significant predictors of achieving the FOTO predicted discharge score. This suggests that the enhanced patient plan of care engagement facilitated by the inclusion of RT inclusion of RTM with in-person care may be a significant differentiator and meaningful adjuvant intervention for improving patient outcomes. The addition of digital support, education, feedback, and encouragement from the navigation staff through the RTM platform, as well as progress tracking through the application, may have contributed to these results. In the model specific to the involved RTM vendor, the care navigation staff provided feedback and insight into the patient’s progress and engagement outside of clinic visits to the treating in-person physical therapist via secure Health Insurance Portability and Accountability Act-compliant emails, and notes inside of the electronic medical record. The support provided by RTM to patients outside of in-person visits may help patients overcome barriers to adhering to PT and their home exercise programs.^7^ Increased adherence and engagement in both in-person PT and home exercise programs may have facilitated improved patient outcomes.

The results of this study are consistent with previous research that suggests digital interventions are effective for a variety of medical conditions, ranging from musculoskeletal, cardiopulmonary, and neurologic conditions, for improving function and quality of life.^28^, ^29^, ^30^, ^31^, ^32^, ^33^ The present study’s finding that patients who used the digital intervention of RTM participated more frequently in their PT care is also consistent with previous studies that have found increased patient adherence as well as improved quality of life and reduced health care spending.^31^, ^32^, ^33^ Seron et al^34^ reviewed 53 systematic reviews and concluded that telerehabilitation may be as effective as in-person care for conditions like osteoarthritis, low back pain, and hip and knee replacements. Although encouraging, research on telerehabilitation and digital health is in its infancy. Outside of the present study, there is a paucity of research regarding the use of mobile health (mHealth) modalities with outpatient rehabilitation settings and its effect on patient engagement and outcomes.^28^, ^29^, ^30^, ^31^, ^32^, ^33^

Notwithstanding, the integration of RTM with in-person PT presents a unique opportunity to meet the patient’s needs in a hybrid model of care that combines the advantages of both clinical approaches. Although the evidence for digital-only care pathways is emerging and has shown promise for persons with MSK conditions, the research is relatively sparse. Furthermore, the isolated approach lacks the physical contact necessary for adequate evaluation, specialized testing, and manual treatment strategies. The inclusion of in-person visits, however, allows for a more comprehensive assessment, the ability to exclude red flags or diagnose movement impairments accurately, and the opportunity to implement appropriate hands-on interventions.^14^ The combination of in-person care and remote monitoring addresses these limitations and offers a more holistic approach to MSK rehabilitation.

The results of this study provide support for the benefit of RTM when combined with in-person PT care. The use of remote tools, virtual support, and communication opportunities between in-clinic appointments can augment the traditional care provided by physical therapists. By leveraging digital technology and remote monitoring, health care providers can extend their reach beyond the clinic, and enhance patient engagement.

Although these initial findings are encouraging, more research is needed to understand the efficacy of PT + RTM model of care. Furthermore, research is needed to understand the possible economic implications of incorporating RTM into the care delivery of PT services. MSK conditions impose a significant burden on the health care system and are one of the largest cost drivers in the US.^35^ Studies demonstrate that early adherence to PT can have a significant effect on downstream savings related to MSK conditions, lessening the need for subsequent surgeries, injections, advanced imaging, emergency room visits, and medications such as opioids.^6^ Further studies are needed to understand if the inclusion of RTM into the traditional PT model further amplifies this statistic.

Partnering with payers to study the effect of RTM to improve access and adherence to therapy will provide insight into the potential savings on the total cost of MSK-related care. By identifying patterns and trends in health care utilization, spending, and the effectiveness of different treatment strategies (including RTM), health care providers, insurers, and policymakers may establish opportunities to optimize care pathways and improve patient outcomes, while reducing unnecessary expenses.

### Study limitations

This study includes several limitations. First, the involved patients had to opt-in to remote therapeutic monitoring, increasing the possibility of selection bias, as the patients who agreed to participate in RTM services could have exhibited higher therapy confidence, been more likely to engage in their care plan, and therefore have an improved chance of achieving the FS benchmark from the outset. There is also potential for enrollment bias, given that technology is a requirement of RTM participation, and research has highlighted demographic differences between those with computer and internet access, and those without.^36^

Additionally, the treatment plans incorporated in-clinic, and the home exercise programs prescribed external to the clinic through the DETA were not controlled in this study. Differences in patients’ respective physical therapists, implemented interventions, and care delivery could introduce bias in the results of patient engagement and outcomes.

It is difficult to apply the results of the study to other anatomical regions, and assume other MSK DX (International Classification of Diseases-10) codes will yield similar results, as the largest case type (back) made up 46% of cases. Furthermore, the authors of this study defined an RTM user as a patient enrolled in RTM as part of their plan of care, who was onboarded onto the DETA, and who completed at least 2 outcomes throughout their course of care, indicating engagement. However, this definition of an RTM user is not uniform throughout the industry. No standardized definition currently exists from the American Medical Association or American Physical Therapy Association, thus potentially skewing data interpretation of the results and ultimately the true effect of RTM between the 2 groups.

## Conclusions

The study investigated the effect of a digital exercise therapy application through a PT + RTM care delivery model on the engagement and clinical outcomes of enrolled patients. The results suggest that including RTM with in-person PT can improve patient engagement and patient-reported outcomes when compared to in-person care only.

These findings have significant implications for optimizing PT delivery and care pathways. Incorporating RTM services with in-person PT care has the potential to enhance patient engagement and treatment effectiveness. The support provided by RTM to patients in between their in-person PT care can help overcome barriers to care, ultimately leading to better outcomes.

### Clinical implications

• Integrating RTM within in-person care may improve patient engagement and treatment effectiveness.
• Use of RTM may improve a patient’s attendance for in-person physical therapy visit.
• Patients who use RTM in addition to in-person care may be more likely to achieve the desired functional improvement.

## Supplier

a. R, version 4.2; The R Project for Statistical Computing.

## Disclosure

M.G., R.L., M.J.G., and N.A. are employees of Limber Health. M.G and N.A. assisted with the transfer of data to Ivy Rehab Network, and all 4 coauthors contributed to the final manuscript. However, they were not involved in the data analysis or interpretation of results, which were conducted by A.G. and T.M. The other authors have nothing to disclose.
